# The effect of transient oxygenation on stem cell mobilization and ischemia/reperfusion heart injury

**DOI:** 10.1371/journal.pone.0192733

**Published:** 2018-02-13

**Authors:** Rintaro Yano, Chiaki Inadomi, Lan Luo, Shinji Goto, Tetsuya Hara, Tao-Sheng Li

**Affiliations:** 1 Department of Anesthesiology, Nagasaki University Graduate School of Biomedical Sciences, 1-7-1 Sakamoto, Nagasaki, Japan; 2 Department of Stem Cell Biology, Atomic Bomb Disease Institute, Nagasaki University, 1-12-4 Sakamoto, Nagasaki, Japan; University of Alabama at Birmingham, UNITED STATES

## Abstract

For general anesthesia, pre-oxygenation is routinely performed prior to intubation. It is well-known that ischemic/hypoxic preconditioning induces stem cell mobilization and protects against ischemia/reperfusion (I/R) injury. In this study, we investigated the effect of transient oxygenation on stem cell mobilization and I/R injury of the heart. Mice were exposed to 100% oxygen for 5 or 20 minutes. We evaluated the number of c-kit^+^ stem/progenitor cells and the levels of SDF-1α and VEGF in peripheral blood at 1, 3, 6, and 24 hours after oxygenation. We also induced I/R injury of the heart at 3 hours post-oxygenation for 5 minutes and then examined stem cell recruitment and fibrotic changes in the heart 3 or 14 days later. The number of c-kit^+^ cells in peripheral blood was significantly increased at 1 or 24 hours after oxygenation for either 5 or 20 minutes. Oxygenation for 5 or 20 minutes did not significantly change the SDF-1α level measured in plasma. However, the plasma VEGF level was decreased at 3 hours post-oxygenation for 20 minutes (p = 0.051). Oxygenation for 5 minutes did not significantly alter the fibrotic area or cell apoptosis. Although oxygenation for 5 minutes increased the number of c-kit^+^ cells in hearts damaged by I/R injury, this difference was not significant between groups due to large variation between individuals (p = 0.14). Although transient oxygenation induces stem cell mobilization, it does not appear to protect against I/R injury of the heart in mice.

## Introduction

Ischemia/reperfusion (I/R) injury of organs, especially the heart, has a major impact on prognosis after major surgery [1, 2]. Oxygenation is thought to be beneficial in the treatment of various pathogenic disorders and may also protect against I/R injury of organs. Thus, pre-oxygenation is commonly performed during general anesthesia and before tracheal intubation. However, there is concern that absorption atelectasis may be caused by the inspiration of 100% oxygen [3].

Hypoxic/ischemic pre-conditioning is an innate phenomenon in which brief exposure to sublethal ischemia provides protection against subsequent I/R injury in various organs [4–6]. Recent studies have reported protection against I/R injury of the heart hours to days after preconditioning [7–10]. We have further demonstrated that the delayed cardioprotection due to ischemic pre-conditioning is associated with the mobilization and recruitment of bone marrow-derived stem cells [11]. In contrast to hypoxia/ischemic stimulation, inspiration of 100% oxygen may increase the oxygen level in blood and induce temporary hyperoxemia. Because pre-oxygenation is routinely performed during general anesthesia, it is critical to know the effect of transient oxygenation on stem cell mobilization and I/R injury of organs.

In this study, we monitored changes in cardioprotective factors and the mobilization of bone marrow-derived stem/progenitor cells in healthy mice after exposure to 100% oxygen for 5 or 20 minutes. We further investigated the effect of transient oxygenation on I/R injury of the heart in mice.

## Materials and methods

### Animals and oxygenation

Specific-pathogen-free (SPF) male C57BL/6 mice (6 or 10 -weeks-old) were used in this study. All animals were purchased from Japan CLEA, Inc. (Tokyo, Japan). Green fluorescent protein (GFP)-transgenic mice were kindly provided by Masaru Okabe [12]. This study was approved by the Institutional Animal Care and Use Committee of Nagasaki University (No. 1406021154–2). All experiments were performed in accordance with institutional and national guidelines. The animals were bred in SPF conditions and were allowed free access to food and water in a temperature-controlled environment with a 12:12-h light-dark cycle.

For oxygenation, 10-week-old mice were placed in an enclosed box continuously perfused with 100% oxygen gas and were kept in the box for 5 or 20 minutes (n = 40 for each group).

### Measurement of circulating c-kit^+^ stem/progenitor cells and cardioprotective factors in blood

The mice were killed by cervical dislocation under deep anesthesia induced by intraperitoneal administration of pentobarbital. Blood samples were collected at 1, 3, 6, and 24 hours after oxygenation (n = 8–10 in each time points). We measured the concentrations of SDF-1α and VEGF in plasma by using mouse SDF-1α and VEGF ELISA kits (Abcam, Cambridge, UK), as previously described in detail [11].

To evaluate the mobilization of c-kit^+^ stem/progenitor cells, peripheral blood mononuclear cells were separated from the blood samples by hemolysis. As described previously [11], the cells were stained with PE-conjugated rat anti-mouse c-kit monoclonal antibody (Thermo Fisher Scientific Inc., Waltham, MA, USA) for 20 minutes. Staining for each respective isotype was used as a negative control. After washing, quantitative flow cytometry analysis was performed using a FACS Calibur instrument (Becton Dickinson, Flanklin Lakes, NJ, USA). The acquired data were analyzed using Cell Quest software (Becton Dickinson).

### Bone marrow transplantation (BMT)

BMT was performed as described previously [13]. Briefly, after lethal irradiation (10 Gy), each 6-week-old C57BL/6 mouse was injected intravenously with 5×10^6^ bone marrow mononuclear cells collected from GFP-transgenic mice (n = 3). These chimeric mice (n = 20) were used for experiments 6–8 weeks after BMT.

### Heart I/R injury

Heart I/R injury was induced in the BMT chimera mice (n = 20), as described previously [11]. Briefly, after general anesthesia induced by intraperitoneal administration of pentobarbital and tracheal intubation with a 20-gauge intravenous catheter, the mice were artificially ventilated with room air (Harvard Apparatus Co) at 80 breaths per minute. We performed a left thoracotomy and occluded the left anterior descending artery for 30 min with an 8–0 polypropylene suture [11, 14], followed by reperfusion (n = 10). As a control, sham thoracotomy and suturing were performed in BMT chimeric mice (n = 10). The mice were kept on a heated plate at 37°C to prevent a decrease in body temperature during and after the operation until the mice had recovered from anesthesia. The mice were also injected intraperitoneally with 1 ml warmed saline immediately after surgery and on the day following surgery. We assessed the health of the mice every other day after surgery by tracking wound healing and body weight.

### Histological analysis

Mice were euthanized at 3 or 14 days after surgery (n = 5 at each time point for both groups). The hearts were harvested, and paraffin sections were used for histological analysis as described previously [14, 15]. Sirius red staining was performed to measure the fibrotic area within the left ventricle (LV) anterior wall using Lumina Vision software (Mitani Corp, Fukui, Japan). Measurements were obtained for at least two separate independent sections of each heart, and the averages were used for statistical analysis.

We determined the number of apoptotic cells in the LV anterior wall 3 days after I/R injury using an Apoptag Red In Situ Detection kit (Merck, Darmstadt, Germany). Sections were stained with DAPI to visualize the nuclei. At least 10 different fields were randomly selected for cell counting from two independent sections of each heart, and the averages were used for statistical analysis.

To estimate the recruitment of bone marrow-derived GFP^+^ cells, paraffin sections were incubated with Alexa Fluor 488-conjugated rabbit anti-GFP polyclonal antibody (Thermo Fisher Scientific Inc.) as described previously [11]. Stem cells were further identified by staining with PE-conjugated rat anti-mouse c-kit monoclonal antibody (Thermo Fisher Scientific, Inc.) [11]. The sections were also stained with DAPI to visualize the nuclei. We counted the GFP^+^ cells and GFP^+^/c-kit^+^ cells present in at least 10 different fields from two independent sections of each heart, and the averages were used for statistical analysis.

### Statistical analyses

All results are presented as the mean ± standard error of the mean (SEM). The statistical significance was determined by one-way analysis of variance followed by the Bonferroni post-hoc test (Dr. SPSS II, Chicago, IL). Differences were considered significant when *P* < 0.05.

## Results

### Transient oxygenation significantly increased the incidence of circulating c-kit^+^ stem/progenitor cells in the blood

**Fig 1:**
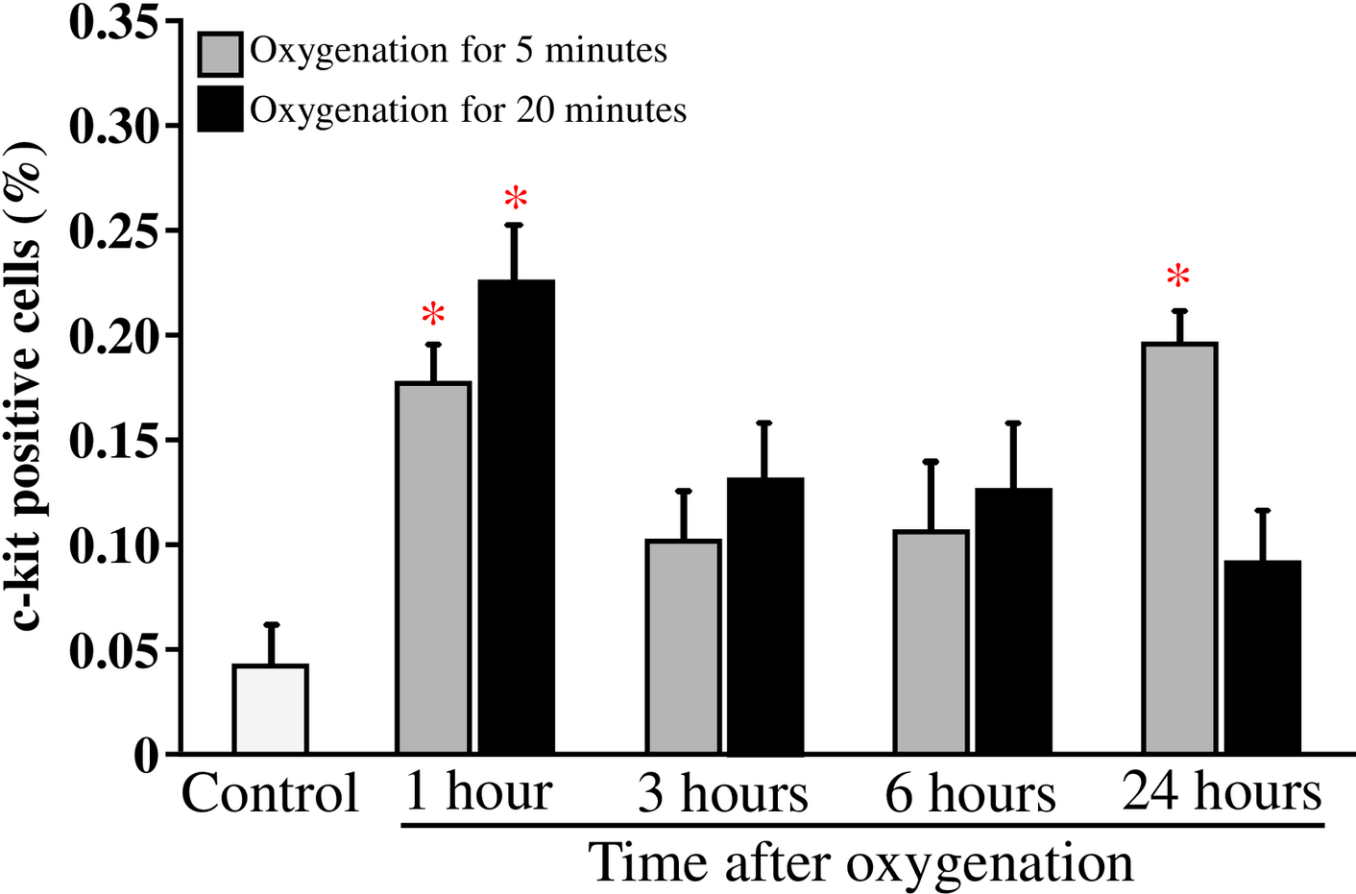
Kinetics of the c-kit^+^ stem/progenitor cells in peripheral blood after oxygenation. Flow cytometry analysis was used to determine the number of circulating c-kit^+^ stem/progenitor cells, and the data at each time point were derived from between 6 and 10 mice. Oxygenation for 5 minutes (open bar, n = 9–10 at each time point); Oxygenation for 20 minutes (solid bar, n = 6–8 at each time point). * P < 0.01 *vs*. Control (n = 9).

When compared to control mice, the number of c-kit^+^ cells in the peripheral blood of mice was significantly increased at 1 and 24 hours, but not at 3 and 6 hours after oxygenation for 5 minutes (Fig 1). Conversely, the number of c-kit^+^ cells in peripheral blood was significantly increased only at 1 hour after oxygenation for 20 minutes (Fig 1).

**Fig 2:**
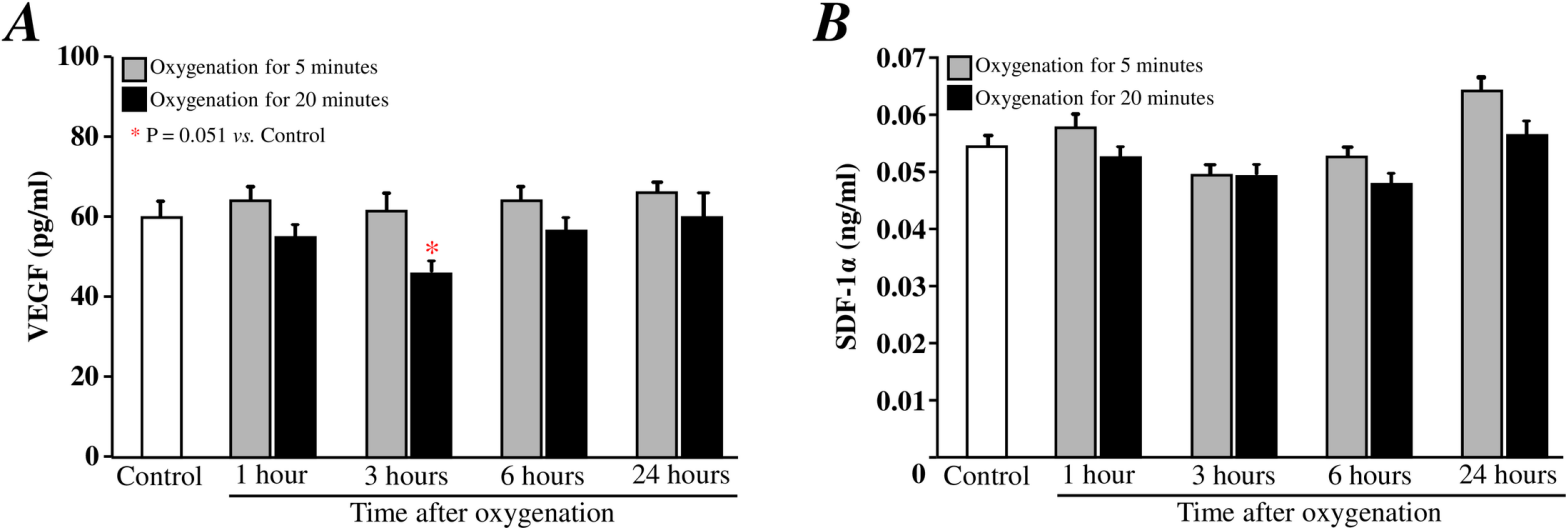
Kinetics of cardioprotective factors in the plasma after oxygenation. ELISA was performed in duplicate to measure the levels of SDF-1α (A) and VEGF (B) in plasma, and the data at each time point were derived from between 5 and 6mice. Oxygenation for 5 minutes (open bar, n = 6 at each time point); Oxygenation for 20 minutes (solid bar, n = 5–6 at each time point).

The level of VEGF in plasma did not change at 1, 3, 6, and 24 hours after oxygenation for 5 minutes (Fig 2A), but trended to decrease at 3 hours after oxygenation for 20 minutes (p = 0.051 *vs*. control; Fig 2A). Compared with the control, the levels of SDF-1α in plasma did not change significantly at 1, 3, 6, or 14 hours after oxygenation for either 5 or 20 minutes (Fig 2B).

### Transient oxygenation had a limited effect on I/R heart injury

**Fig 3:**
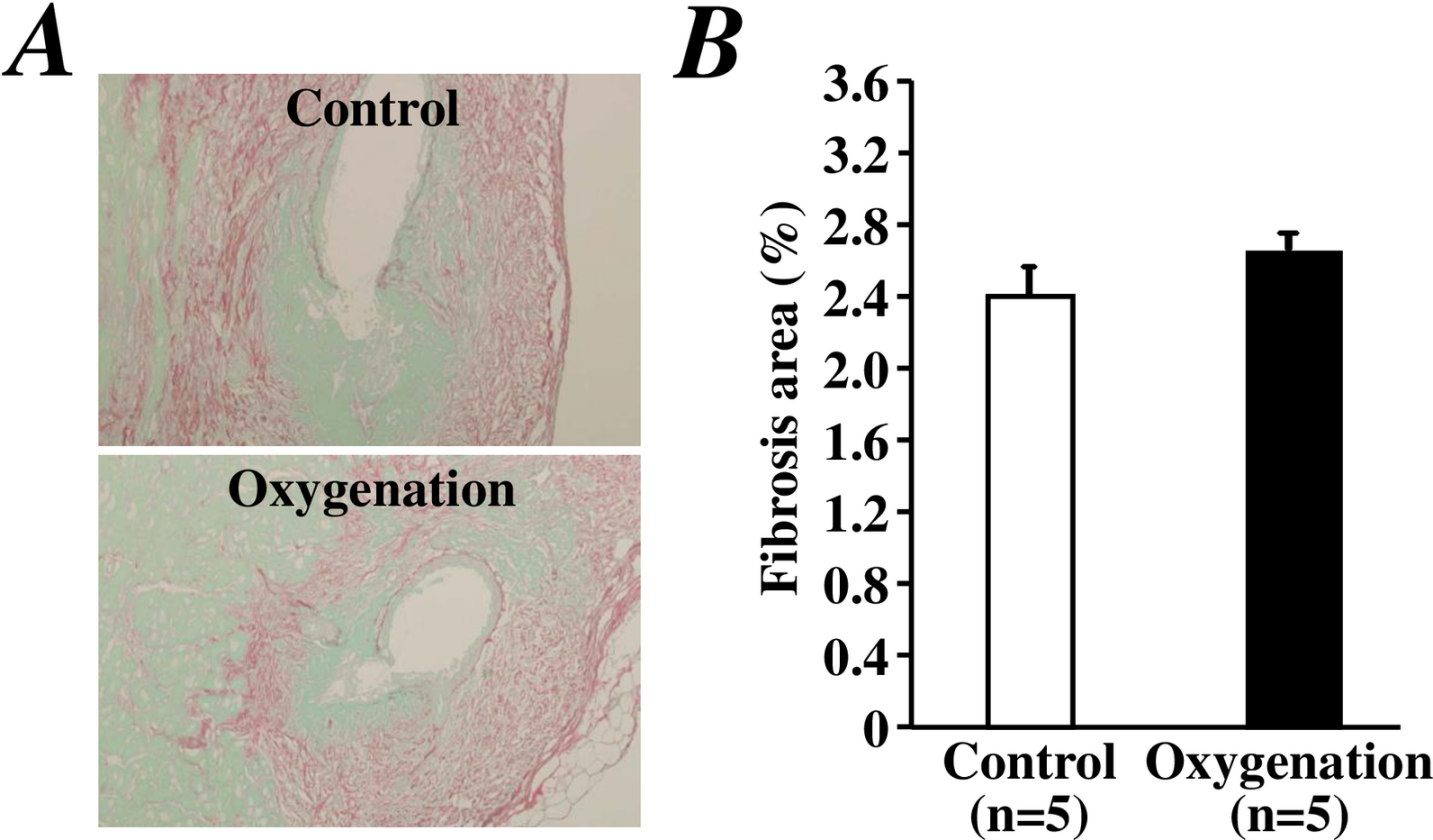
Sirius red staining for fibrotic tissue 2 weeks after I/R injury of the heart. Representative images (A) and quantitative data (B) of the fibrotic area within the LV anterior wall are shown as a percentage of total cross section area.

Sirius red staining clearly detected fibrotic tissue in the LV anterior wall 14 days after I/R injury in both groups (Fig 3A). Quantitative analysis revealed that there was not a significantly difference in the fibrotic area of the LV anterior wall between groups (Fig 3B).

**Fig 4:**
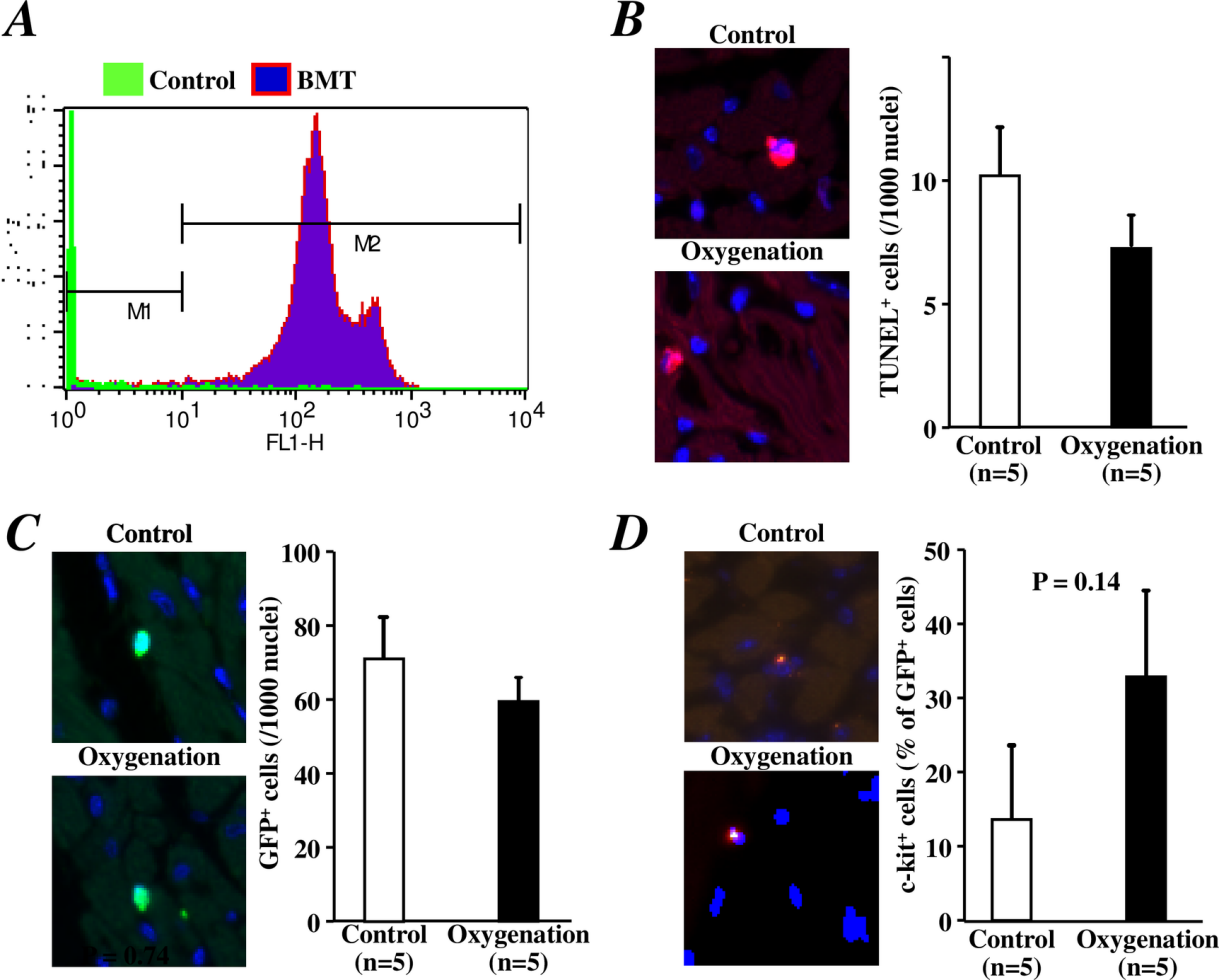
Histological analyses of cell apoptosis and the recruitment of bone marrow-derived (stem) cells 3 days after I/R injury of the heart. A) A representative histogram of flow cytometry revealed that over 90% of circulating blood cells were replaced by GFP-positive cells at 6 weeks after post-transplantation with bone marrow cells from GFP-transgenic mice. B) Representative images (left) and quantitative data (right) of TUNEL-positive apoptotic cells in the LV anterior wall of the heart are shown. C) Representative images (left) and quantitative data (right) of bone marrow-derived GFP^+^ cells in the LV anterior wall of the heart are shown. D) Representative images (left) and quantitative data (right) of GFP^+^/c-kit^+^ stem cells in the LV anterior wall of the heart are shown.

At 6 weeks after BMT, greater than 90% of the circulating blood cells in the chimeric mice were GFP-positive (Fig 4A). Although TUNEL staining analysis revealed fewer apoptotic cells in the LV anterior wall in the oxygenation group than in the control group at 3 days after I/R injury, the difference between the groups was not significant (Fig 4B). The GFP^+^ cells (Fig 4C) and GFP^+^/c-kit^+^ cells (Fig 4D) were more frequently observed in the LV anterior wall than in other areas unaffected by I/R injury. By counting the total number of bone marrow-derived GFP^+^ cells and GFP^+^/c-kit^+^ cells within the LV anterior wall at 3 days post-I/R injury, we found that the number of GFP^+^ cells and GFP^+^/c-kit^+^ cells did not differ significantly between the groups (Fig 4C). However, more GFP^+^/c-kit^+^ cells were counted in the oxygenation group than in the control group (P = 0.14, Fig 4D).

## Discussion

I/R injury of organs is one of the major complications of surgical procedures. Stem cells play a critical role in organ homeostasis, including physiological turnover and pathological regeneration after injury. Hypoxic/ischemic stimulation has been shown to induce stem cell mobilization and to protect against I/R injury of organs [16–21]. We therefore speculate that the inspiration of 100% oxygen, a procedure routinely performed before intubation during general anesthesia, may induce temporary hyperoxemia, which could inhibit the mobilization and recruitment of stem cells and even sensitize the patient to I/R injury.

We first investigated the mobilization of stem cells and changes in the levels of VEGF and SDF-1α in the peripheral blood of young healthy mice after transient oxygenation. Our data showed that exposure to 100% oxygen for either 5 or 20 minutes significantly increased the number of circulating c-kit^+^ stem/progenitor cells in peripheral blood after as little as 1 hour. An increased number of stem cells was also observed 24 hours after oxygenation for 5 minutes. However, the SDF-1α and VEGF levels in plasma were not significantly increased by oxygenation for either 5 or 20 minutes. In our previously study, we found that ischemic preconditioning mobilizes stem cells during the late phase (24 hours) but not during the early phase (1–6 hours) [11]. In contrast, ischemic preconditioning increases the VEGF and SDF-1α levels in plasma at a very early time point (1 hour), but these levels return to normal within 6 hours [11]. Hypoxic and ischemic stimulations are well known to induce HIF-1alpha, which in turn promotes the production of VEGF [22]. The mobilization of stem cells by hypoxic/ischemic stimulation is also well characterized [23]. In contrast, the effect of oxygenation on stem cell mobilization is largely unknown. Hyperoxemia has been reported to inhibit the HIF-1 pathway [24], which may result in the inhibition of VEGF production. Indeed, the VEGF level in plasma was decreased at 3 hours post-oxygenation for 20 minutes. Interestingly, oxygenation for 5 minutes did not alter VEGF levels. Because we did not measure oxygen saturation, it is unclear whether hyperoxemia was induced by a longer oxygenation (20 minutes) but not by the shorter oxygenation (5 minutes). Furthermore, we found the number of c-kit^+^ stem/progenitor cells in peripheral blood increase at 24 hrs after oxygenation for 5 minutes but not for 20 minutes. Without an increase in SDF-1α and VEGF levels in plasma, it remains a mystery how transient oxygenation induces the mobilization of c-kit^+^ stem/progenitor cells. It is critical to know whether temporary hyperoxemia and a transient decrease in VEGF play roles in the mobilization of c-kit^+^ stem/progenitor cells. Further experiments, such as measuring the levels of erythropoietin and other factors in plasma and the oxygen tension in bone marrow, must be performed to uncover the mechanisms of stem cell mobilization after oxygenation.

Because the mobilization of stem/progenitor cells has been demonstrated to protect against I/R injury of the heart during the late phase (24 hours) after ischemic preconditioning [11], we investigated the effect of transient oxygenation on I/R heart injury. We used the same I/R injury model published in a previous study [11]. Unexpectedly, our data showed that oxygenation for 5 minutes did not attenuate I/R heart injury, although the number of circulating c-kit^+^ stem/progenitor cells in peripheral blood was clearly increased within 24 hours after oxygenation for 5 minutes. The reason for the difference between these studies remains unclear. We speculate that without the increase in SDF-1α and VEGF after oxygenation, the recruitment of a small number of c-kit^+^ cells is insufficient to effectively protect against I/R injury of the heart.

This study has several limitations. First, we did not attempt to uncover the mechanism underlying stem cell mobilization after oxygenation. Second, we only investigated the protective effect against I/R injury of the heart after oxygenation for 5 minutes; it is unclear whether longer exposure to oxygen would sensitize I/R injury of the heart or other organs. Third, we only measured c-kit^+^ cells within the LV anterior wall. Although there is no consensus at present regarding definitive markers of cardiac stem cells, several factors, including islet-1 and Sca-1, are frequently used for their identification [25]. Finally, while young healthy mice were used for study, clinical oxygenation is usually given to aged patients. It would be interesting to determine whether the response to oxygenation differs between physiological and pathological conditions.

In conclusion, our preliminary data showed that transient oxygenation induced, rather than inhibited, the mobilization of bone marrow-derived stem/progenitor cells. However, 5 minutes of oxygenation had a very limited effect on I/R injury of the heart. Although it is not clear whether oxygenation is toxic after a long period time, transient (<20 minutes) inspiration of 100% oxygen during general anesthesia for surgical operations appears to be safe and has a limited effect on I/R injury of organs.
